# Association of Parental and Contextual Stressors With Child Screen Exposure and Child Screen Exposure Combined With Feeding

**DOI:** 10.1001/jamanetworkopen.2019.20557

**Published:** 2020-02-05

**Authors:** Katherine Tombeau Cost, Daphne Korczak, Alice Charach, Catherine Birken, Jonathon L. Maguire, Patricia C. Parkin, Peter Szatmari

**Affiliations:** 1 Department of Psychiatry, The Hospital for Sick Children, Toronto, Ontario, Canada; 2 Department of Psychiatry, Faculty of Medicine, University of Toronto, Toronto, Ontario, Canada; 3 Division of Paediatric Medicine, The Hospital for Sick Children, Toronto, Ontario, Canada; 4 Department of Pediatrics, Faculty of Medicine, University of Toronto, Toronto, Ontario, Canada; 5 Sick Kids Research Institute, Toronto, Ontario, Canada; 6 Institute of Health Policy, Management and Evaluation, University of Toronto, Toronto, Ontario, Canada; 7 Department of Nutritional Sciences, Faculty of Medicine, University of Toronto, Toronto, Ontario, Canada; 8 Li Ka Shing Knowledge Institute, St Michael’s Hospital, Toronto, Ontario, Canada; 9 Department of Pediatrics, St Michael’s Hospital, University of Toronto, Toronto, Ontario, Canada; 10 Paediatric Outcomes Research Team, Division of Paediatric Medicine, The Hospital for Sick Children, Toronto, Ontario, Canada; 11 Centre for Addiction and Mental Health, Department of Psychiatry, The Hospital for Sick Children, University of Toronto, Toronto, Ontario, Canada

## Abstract

**Importance:**

Parents may use screen exposure or screen exposure combined with feeding for infants and toddlers to alleviate their own stress.

**Objective:**

To evaluate the association of individual and contextual stressors with child screen exposure (CSE) and CSE combined with feeding (CSE+F) in children aged 7 to 18 months as measured by parent-reported use and variation in quantity of CSE and CSE+F.

**Design, Setting, and Participants:**

This cross-sectional, population-based study drew parent-child participants from TARGet Kids, a network of primary health care research settings throughout Toronto, Ontario, Canada, between November 1, 2011, and July 31, 2018. The included children were aged 7 to 18 months. Exclusion criteria were health conditions affecting growth, acute conditions, chronic conditions, severe developmental delay, and families who were unable to communicate in the English language. Data were analyzed from April 1, 2019, to July 31, 2019.

**Exposures:**

Parenting stress, child age, child negative affectivity, family living arrangements, and family income were assessed with parent-completed questionnaires.

**Main Outcomes and Measures:**

The primary outcome was CSE and the variation in CSE, which represented the amount of background and engaged use of screens (television, videos or DVDs, video games, computers, and mobile devices) in a typical week. The secondary outcome was CSE+F, which represented the frequency of breakfast, lunch, dinner, and snack consumption during screen exposure in a typical week. Both CSE and CSE+F were assessed with the parent-completed Nutrition and Health Questionnaire; Infant Behavior Questionnaire, Revised Very Short; and Parenting Stress Index, Short Form.

**Results:**

The final sample size was 1085 children for the analysis of CSE and 1083 children for the analysis of CSE+F. Among 914 respondents, the children’s mean (SD) age was 11.6 (2.3) months, and 478 (52.3%) were male. Among 910 respondents, 839 (92.2%) were mothers, with a mean (SD) age among 873 respondents of 34.4 (4.2) years. In a typical week, 581 of 779 children (74.6%) were reported to have any CSE, and 321 of 874 children (36.7%) had some CSE+F. Higher parenting stress levels (odds ratio [OR], 1.01; 95% CI, 1.00-1.02; *P* = .04) and older child age (OR, 1.43; 95% CI, 1.01-2.03; *P* = .04) were associated with CSE. Higher parenting stress levels (incidence rate [IR], 1.00; 95% CI, 1.00-1.01; *P* = .002) and lower household income (IR, 1.26; 95% CI, 1.10-1.45; *P* = .01) were associated with increased CSE. Older child age (OR, 1.79; 95% CI, 1.35-2.38; *P* < .001) and lower household income (OR, 2.54; 95% CI, 1.72-3.74; *P* < .001) were associated with CSE+F. Lower household income (IR, 1.21; 95% CI, 1.03-1.42; *P* = .02) was associated with increased CSE+F.

**Conclusions and Relevance:**

This study found that parenting stress, income, and child age were associated with CSE, and child age and household income were associated with CSE+F. Given these findings, interventions to improve parental coping mechanisms may decrease child screen exposure.

## Key Points

**Question:**

Are individual and contextual stressors associated with the use and duration of screen time and screen time combined with food in children aged 7 to 18 months?

**Findings:**

In this cross-sectional, population-based study of 1085 children, higher levels of parenting stress and lower household income were associated with increased child screen exposure, whereas higher parenting stress levels and older child age, but not income, were associated with child screen exposure. Only lower household income was associated with increased child screen exposure with feeding, whereas both lower household income and older child age were associated with child screen exposure with feeding.

**Meaning:**

Findings of this study suggest that individual stressors, including higher parenting stress, and contextual stressors, including lower household income, were associated with pervasive screen exposure variables in children aged 7 to 18 months.

## Teaser

This population-based cross-sectional study examines the associations between parental, child, and household factors with the amount and frequency of electronic screen time and screen time with feeding among infants and toddlers in Toronto, Ontario, Canada.

## Introduction

The risks, benefits, and variables associated with child screen exposure (CSE), defined as time passively spent in the presence of screens and/or time actively spent viewing or engaging with screens (eg, television, videos or DVDs, video games, computers, and mobile devices), are areas of interest for researchers and of concern for parents.^1^ Child age, duration of exposure, screen content, meals or snacks consumed during exposure, and the conditions under which screen exposure is accepted or encouraged are all contested topics among parents and child development experts. Child screen exposure and CSE combined with feeding (CSE+F; defined as screen time exposure while eating meals or snacks) are influenced by parenting beliefs, such as the educational value of screen time, and contextual factors, such as availability of screens in the home.^2,3,4^ Some studies have found positive outcomes of video chatting in younger children^5^ or differential effects of screen modality.^6^ However, other studies report that use of mobile devices by children younger than 2 years is associated with shorter sleep duration.^7^ Furthermore, results are mixed as to whether, when, or how video chatting is advantageous for children younger than 18 months.^8,9,10,11^ Across several studies, CSE among infants and toddlers has been associated with developmental problems^12^ spanning several domains, including socioemotional,^13^ cognitive,^14^ and language.^15^ It is, therefore, imperative to understand the factors associated with early CSE and CSE+F to develop effective interventions to prevent negative outcomes.

### Variables Associated With Screen Exposure

Demographic variables associated with increased CSE include child age and race/ethnicity, specifically older age and minority status.^16^ Associations between early CSE and maternal age, parental educational level, and household income have been mixed, with lower parental educational level and lower household income found to be associated with increased CSE in some^17,18^ but not all studies.^16^ In addition, the home media environment, which considers screen time rules, accessibility, and parent modeling, has been associated with the amount of CSE.^19^ Parents’ use of screen time rules and limits, combined with their greater self-efficacy in creating and enforcing those rules, has been associated with decreased CSE.^20^

Parental cognitive and psychological states have also been associated with CSE. Low parental health literacy was associated with increased CSE among children in the first 2 years of life.^21^ Maternal beliefs that CSE was advantageous or educational for their child were also associated with increased CSE in infants.^16^ Greater maternal distress was associated with increased CSE.^22^ Lower child self-regulation, an aspect of temperament that can be a stressor to parents, was associated with increased CSE at 2 years of age.^23^

### Screen Exposure Combined With Feeding in Younger Children

Child screen exposure combined with feeding has been associated with a lower-quality diet, such as increased intake of sugar-sweetened beverages and high-sugar and high-fat foods as well as decreased intake of fruits and vegetables^24^ and also with increased adolescent adiposity and higher insulin resistance.^25^ Variables associated with CSE+F may be different from variables associated with CSE, but the literature on CSE+F is more limited than that on CSE. Demographic variables associated with increased CSE+F in the first 4 years of life were income less than US $50 000 per year and fathers with less than a college degree.^18^ Lower maternal educational level was also associated with increased CSE+F.^26^ Household environment variables associated with CSE+F included a television in the child’s bedroom.^26^ Child temperament, including higher infant activity at age 9 months,^26^ and greater negative affectivity in older children^27^ were also associated with increased CSE+F. Data on CSE and findings from these studies indicate that contextual and personal stressors may propel parents to use CSE or CSE+F as a coping mechanism or peacekeeper^3,28^ despite recommendations against it in children of this age group.^29^

### Evidence Gaps

Fewer studies have been performed of individual stressors, such as parenting stress, child temperament, and child mobility and autonomy, and contextual stressors, such as family composition and household income, as variables associated with CSE and CSE+F that may indicate the use of screen time as a parental coping mechanism.^3,30^ Moreover, the stressors that are associated with the use of CSE and CSE+F may differ from the stressors that are associated with the increased amount or frequency of CSE and CSE+F. Given these gaps in the literature, we explored the association between individual and contextual stressors and CSE and CSE+F in children aged 7 to 18 months. Our primary hypothesis was that higher stress levels, measured by parenting stress, child negative affectivity, greater child mobility and autonomy with older age, household composition, and household income, would be associated with the use of CSE and CSE+F in children aged 7 to 18 months as well as variations in those 2 variables.

## Methods

### Study Design and Sample Population

A cross-sectional design was used for the present study, with each parent-child dyad represented only once in the data set. Participants were drawn from the TARGet Kids primary care practice–based research network throughout the city of Toronto, Ontario, Canada. Healthy children aged 7 to 18 months were enrolled and followed up through scheduled health supervision visits. Exclusion criteria were health conditions affecting growth, acute conditions, chronic conditions, severe developmental delay, and inability to communicate in the English language. Data used in these analyses were collected from November 1, 2011, through July 31, 2018. Ethics approval was obtained from The Hospital for Sick Children. All participants provided written informed consent before enrollment in the study. We followed the Strengthening the Reporting of Observational Studies in Epidemiology (STROBE) reporting guideline.^31^

All parent and child demographic information was assessed using the standardized parent-report questionnaires,^32^ including parent relationship to child; household living arrangements; maternal age, race/ethnicity, educational level, and employment status; self-reported household income; other children in the home; and child age (2 age groups: 7-12 months and 13-18 months) and sex.

### Infant Behavior Questionnaire and Parenting Stress Index

Child negative affectivity was assessed with the negative affectivity subscale (score range: 1.20-6.83, with a higher score indicating greater negative affectivity) of the Infant Behavior Questionnaire, Revised Very Short, a 36-item, 7-point Likert scale questionnaire for evaluating child temperament. The psychometric properties of this subscale are good, with excellent internal consistency (Cronbach α = 0.91), good construct validity, and good interrater reliability from secondary caregivers (Cronbach α = 0.70).^33^ The negative affectivity scale had good internal consistency in the present sample (Cronbach α = 0.84).

Parenting stress was assessed with the Parenting Stress Index, Short Form (PSI-SF), a 36-item, parent-reported questionnaire completed when the child was aged 7 to 18 months. The PSI-SF (score range: 38-104, with a higher score indicating more parenting stress) has 3 subscales (parental distress, parent-child dysfunctional relationship, and difficult child) that are combined for a total score. The PSI-SF has good psychometric properties, with acceptable test-retest reliability (0.78 intraclass correlation), good internal consistency (Cronbach α = 0.85), good construct validity, and good differentiation between at-risk and not-at-risk groups.^34^ The PSI-SF total score had excellent internal consistency in the present sample (Cronbach α = 0.92).

### CSE and CSE+F 

Child screen exposure and CSE+F were assessed using the Nutrition and Health Questionnaire, completed by the parent at the well-child visit. We created the primary outcome CSE total score to represent the amount of time the child spent with a screen on during a typical week, and we posed the following questions: (1) “On a typical weekday, how many minutes did your child spend awake in a room with television on? Videos or a DVD on? Playing the computer? Playing video game consoles? Playing handheld devices?” and (2) “On a typical weekend day, how many minutes did your child spend awake in a room with television on? Videos or a DVD on? Playing the computer? Playing video game consoles? Playing handheld devices?” We multiplied the sum for each type of screen time in the first set of questions by 5 and summed the products. We multiplied the total for each type of screen time in the second set of questions by 2 and summed the products. To compute the sum of the total minutes of screen time in a typical week, we added the total of weekday minutes and the total of weekend minutes of screen time.

We created the secondary outcome CSE+F total score to represent the frequency of CSE combined with feeding using these questions: (1) “On a typical weekday, which meals did your child eat in a room with a screen device on (television, computer, tablet, etc): Breakfast? Lunch? Snack? Dinner?” and (2) “On a typical weekend day, which meals did your child eat in a room with a screen device on (television, computer, tablet, etc): Breakfast? Lunch? Snack? Dinner?” Similar to the procedure for creating the CSE variable, we added the sum of weekday screen time with feeding and the sum of weekend screen time with feeding.

### Statistical Analysis

All unique cases for cross-sectional analysis were identified by completion of any question on the PSI-SF. Using the 3σ rule, we identified 2 outliers in the PSI-SF, 16 outliers on CSE, and 0 outliers in negative affectivity or CSE+F. To reduce the loss of data, we rescaled the outliers using the convert back from *Z* score method^35^ in PSI-SF and CSE to be equal to 3 SDs above the mean for each measure. All results were the same whether outliers were retained or rescaled.

To address all of the research questions on the linear count increases in CSE and CSE+F as well as the logistic use or nonuse of CSE and CSE+F at age 7 to 18 months, we used zero-inflated negative binomial regression with the pscl package and created plots with the ggplot2^36^ package in R (R Project for Statistical Computing). To address the research questions on CSE, we used zero-inflated negative binomial regression to identify the variables associated with CSE compared with no CSE at 7 to 18 months of age as well as the variables associated with increased CSE among children with any screen time at 7 to 18 months. We used an identical zero-inflated negative binomial regression procedure with CSE+F as an outcome. Again, this method permitted simultaneous logistic associations for CSE+F compared with no CSE+F at age 7 to 18 months and also for increased CSE+F among children with any CSE+F.

We compared the characteristics of participants with missing data with those with complete data (eTable 1 in the Supplement). To reduce the loss of cases to missing data, we used the mice package^37^ in R for multiple imputation of missing data (n = 50). We followed the premise that the number of imputations should be similar to the percentage of cases that are incomplete.^38^ We imputed item-level data when possible to increase power and entered all of the information available to impute the missing values.^39^ We then applied the item-level imputations to passively impute the total PSI-SF score.^40^

Sample size calculations to detect a small effect (*ƒ*^2^*_B_* = 0.02; power = 0.8; α = .05) with 5 independent variables (parenting stress, child negative affectivity, child age, household composition, and household income) were performed with a priori power analysis using the pwr package^41^ in R. The minimum sample size was determined to be 629.

A 2-sided, unpaired, zero-inflated, negative binomial regression with *P* = .05 indicated statistical significance. Data were analyzed from April 1, 2019, to July 31, 2019.

## Results

### Demographic Characteristics

A total of 1115 participants responded to questions on the PSI-SF questionnaire when their child was between 7 and 18 months of age at the time of analysis. Not all questions were answered by all respondents. The final sample size was 1085 children for the analysis of CSE and 1083 children for the analysis of CSE+F. Among 911 children, 859 (94.3%) lived in a 2-parent household. Among 910 respondents, 839 (92.2%) were mothers, with a mean (SD) age of 34.4 (4.2) years reported by 837. Five hundred ten of 827 mothers (61.7%) reported European ancestry, 767 of 867 overall respondents (88.5%) reported a household income greater than Canadian $60 000 per year (US $45 906), 854 of 906 mothers (94.3%) reported completing college or university, 737 of 884 mothers (83.4%) reported being employed outside of the home, and 523 of 911 overall respondents (57.4%) had only 1 child in the home. Among the children, 478 of 914 (52.3%) were identified as male. The mean (SD) age of the children in the study was 11.6 (2.3) months as reported by 914 respondents. All demographic characteristics are summarized in Table 1, and sample selection is shown in the Figure. A correlation matrix with all demographic and study variables is available in eTable 2 in the Supplement.

**Table 1.** Sample Descriptive Statistics

Characteristic	Total Respondents, No.	No. (%)
Family living arrangements		
2 Parents in the same household	911	859 (94.3)
Single parent or other type of household	911	52 (5.7)
Relationship to child^a^		
Maternal	910	839 (92.2)
Paternal	910	71 (7.9)
Maternal race/ethnicity		
European ancestry	827	510 (61.7)
Non-European ancestry	827	317 (38.3)
Paternal race/ethnicity		
European ancestry	948	583 (61.5)
Non-European ancestry	948	365 (38.5)
Age, mean (SD), y		
Mother^b^	873	34.39 (4.2)
Father^c^	999	35.71 (5.3)
Self-reported household income, CAD $/y		
>60 000	867	767 (88.5)
≤60 000	867	100 (11.5)
Maternal educational level		
College education	906	854 (94.3)
Less than college education	906	52 (5.7)
Maternal employment status		
Employed full-time	884	737 (83.4)
Not employed full-time	884	147 (16.6)
Paternal employment status		
Employed full-time	953	880 (92.3)
Not employed full-time	953	73 (7.7)
Child in licensed day care	1058	177 (16.7)
Other children in the home		
Only child	911	523 (57.4)
>1 Child	911	388 (42.6)
Child age, mean (SD), y	914	11.6 (2.3)
Child sex		
Male	914	478 (52.3)
Female	914	436 (47.7)
IBQ-RVS negative affectivity score, mean (SD)^d^	898	4.30 (1.0)
No. of CSE+F instances/wk, mean (SD)^e^	874	4.4 (7.4)
CSE+F/wk		
None	874	553 (63.3)
Any	874	321 (36.7)
Total CSE/wk, mean (SD), min^f^	779	415.3 (527.7)
CSE/wk		
None	779	198 (25.4)
Any^g^	779	581 (74.6)
Parenting Stress Index, Short Form total score, mean (SD)^h^	914	59.35 (14.3)

Abbreviation: CAD, Canadian; CSE, child screen exposure; CSE+F, child screen exposure combined with feeding; IBQ-RVS, Infant Behavior Questionnaire, Revised Very Short.

^a^ Includes biological, adoptive, and stepparent.

^b^ Range of maternal age, 21 to 53 years.

^c^ Range of paternal age, 19 to 55 years.

^d^ Range of negative affectivity scores, 1.20 to 6.83, with a higher score indicating greater negative affectivity.

^e^ Range of CSE+F instances per week, 0 to 28.

^f^ Range of CSE duration per week, 0 to 2289 minutes.

^g^ Includes only participants who answered all questions on CSE, including all modalities.

^h^ Range of Parenting Stress Index, Short Form total scores, 38 to 104, with a higher score indicating more parenting stress.

**Figure.**
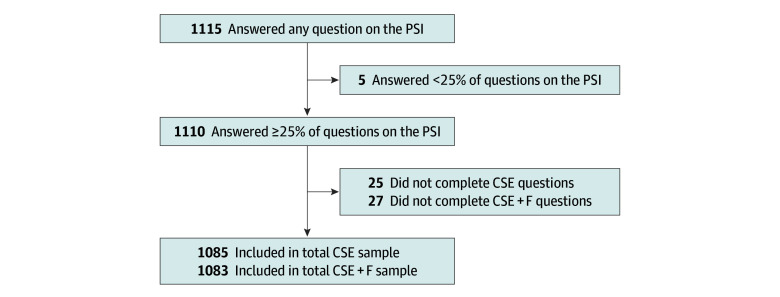
Sample Selection Flowchart CSE indicates child screen exposure; CSE+F, child screen exposure combined with feeding; and PSI, Parenting Stress Index.

### Screen Time and Screen Time Combined With Feeding

In a typical week, 581 of 779 children (74.6%) were reported to have any CSE, whereas 198 respondents (25.4%) reported no CSE at all for their children. The mean (SD) duration of CSE was 415.3 (527.7) minutes (or 6.9 hours) per week. Median (interquartile range [IQR]) duration of CSE was 225.0 (542.0) minutes (or 3.9 [9.03] hours) per week. Furthermore, 321 parents (36.7%) reported any CSE+F, with a mean (SD) occurrence of CSE+F of 4.4 (7.4) meals or snacks per week. The occurrence of CSE+F had a large range, from 0 to 28 meals or snacks during screen time in a week. Means, standard deviations, and binary percentages are shown in Table 1. A detailed comparison of CSE and no CSE by screen type and by year is provided in eTable 3 in the Supplement.

The logistic component of the model for CSE demonstrated that older children were more likely to have any CSE (odds ratio [OR], 1.43; 95% CI, 1.01-2.03; *P* = .04) (Table 2) compared with younger children. A 1-unit increase in the child age category (ie, from 7-12 months to 13-18 months) was associated with a 43% increase in the odds of any reported CSE. Among children aged 7 to 12 months, 472 of 648 (72.8%) had CSE, but among children aged 13 to 18 months, only 207 of 253 (81.8%) had CSE. Children whose parents reported higher parenting stress levels (PSI-SF score) were more likely to have any CSE (OR, 1.01; 95% CI, 1.00-1.02; *P* = .04) (Table 2). A 1-SD increase (14.3 points) in PSI-SF score was associated with a 17% decrease in the odds of no reported CSE. Among children with no CSE, the mean (SD) level of parenting stress was 59.3 (1.1), whereas for children with any CSE, the mean (SD) level of parenting stress was 62.0 (0.6). Under the linear count component of the model for CSE, among children with any CSE, as parenting stress increased, the amount of CSE also increased (incidence rate [IR], 1.00; 95% CI, 1.00-1.01; *P* = .002) (Table 2). A 1-SD increase (14.3 points) in parenting stress was associated with a 6% increase in the number of minutes of CSE. For the average parent in the present study, a 1-SD increase in parenting stress translated into an additional 24.9 minutes of CSE per week. Among children with any CSE, as income decreased from more than $60 000 per year to $60 000 or less per year, the amount of CSE increased (IR, 1.26; 95% CI, 1.10-1.45; *P* = .01) (Table 2). A 1-unit decrease in household income category (ie, from >$60 000 per year to ≤$60 000 per year) was associated with a 26% increase in the number of CSE minutes. For the average parent in this study, being in a low-income category compared with being in a high-income category translated into 108.0 additional minutes of CSE per week.

**Table 2.** Zero-Inflated Negative Binomial Regression Variables Associated With Child Screen Exposure Among 1085 Children

Variable	No CSE vs Any CSE^a^		CSE Count^b^	
	OR (95% CI)	*P* Value	IR (95% CI)	*P* Value
Unadjusted model				
Parenting Stress Index, Short Form total score	1.01 (1.00-1.02)	.01	1.00 (1.00-1.01)	.009
Adjusted model				
Parenting Stress Index, Short Form total score	1.01 (1.00-1.02)	.04	1.00 (1.00-1.01)	.002
Child age	1.43 (1.01-2.03)	.04	1.05 (0.99-1.12)	.09
Negative affectivity score	1.07 (0.92-1.25)	.34	1.00 (0.96-1.03)	.76
Self-reported household income	1.42 (0.86-2.33)	.17	1.26 (1.10-1.45)	.01
Living arrangements	1.32 (0.62-2.82)	.47	1.05 (0.91-1.22)	.45

Abbreviations: CSE, child screen exposure; IR, incidence rate; OR, odds ratio.

^a^ Indicates variables associated with the transition from no CSE to any CSE.

^b^ Indicates variables associated with increased CSE if CSE is being used.

For the CSE+F analysis, under the logistic component of the model, older children were more likely to have any CSE+F compared with younger children (OR, 1.79; 95% CI, 1.35-2.38; *P* < .001) (Table 3). A 1-unit increase in child age category (ie, from 7-12 months to 13-18 months) was associated with a 79% increase in the odds of having any CSE+F. Among children aged 7 to 12 months, 243 of 733 (33.2%) had CSE+F, but among children aged 13 to 18 months, 145 of 296 (49.0%) had CSE+F. Children in lower-income families (≤$60 000 per year) were more likely to have any CSE+F (OR, 2.54; 95% CI, 1.72-3.74; *P* < .001) (Table 3) compared with children in higher-income families (>$60 000 per year). A 1-unit decrease in household income category (ie, from >$60 000 per year to ≤$60 000 per year) was associated with a 154% increase in the odds of having any CSE+F. Among children in higher-income families, 284 of 845 (33.6%) had CSE+F, but among children in lower-income families, 73 of 129 (56.6%) had CSE+F. Under the linear count component of the model for CSE+F, among children with any CSE+F, as income decreased, the frequency of CSE+F increased (IR, 1.21; 95% CI, 1.03-1.42; *P* = .02; Table 3). A 1-unit decrease in household income category (ie, from >$60 000 per year to ≤$60 000 per year) was associated with a 21% increase in the frequency of CSE+F. For the average parent in this study, being in a low-income category compared with being in a high-income category translated into 0.9 more instances of CSE+F per week.

**Table 3.** Zero-Inflated Negative Binomial Regression Variables Associated With Child Screen Exposure Combined With Feeding Among 1083 Children

Variable	No CSE+F vs Any CSE+F^a^		CSE+F Count^b^	
	OR (95% CI)	*P* Value	IR (95% CI)	*P* Value
Unadjusted model				
Parenting Stress Index, Short Form total score	1.01 (1.00-1.01)	.25	1.00 (1.00-1.01)	.11
Adjusted model				
Parenting Stress Index, Short Form total score	1.00 (0.99-1.01)	.62	1.00 (1.00-1.01)	.21
Child age	1.79 (1.35-2.38)	<.001	1.00 (0.87-1.14)	.99
Negative affectivity score	1.07 (0.94-1.23)	.31	1.02 (0.96-1.10)	.48
Self-reported household income	2.54 (1.72-3.74)	<.001	1.21 (1.03-1.42)	.02
Living arrangements	0.83 (0.48-1.44)	.51	0.94 (0.73-1.20)	.62

Abbreviations: CSE+F, child screen exposure combined with feeding; IR, incidence rate; OR, odds ratio.

^a^ Indicates variables associated with the transition from no CSE+F to any CSE+F.

^b^ Indicates variables associated with increased CSE+F if CSE+F is being used.

## Discussion

In this cross-sectional study, approximately three-quarters of children aged 7 to 18 months were exposed to screens daily, and one-third had screen exposure combined with feeding. The mean screen time was just under 1 hour per day. Both individual and contextual stressors were associated with increased CSE; higher parenting stress levels and lower household income were associated with an increasing amount of CSE. In contrast, developmental factors and individual stressors were associated with a greater likelihood of CSE; older child age and greater parenting stress were associated with occurrence of CSE compared with no CSE by age 7 to 18 months. Only the contextual stressor of lower household income was associated with increased frequency of CSE+F. However, both contextual stressors and developmental factors were associated with the use of CSE+F; lower household income and older child age (attended by increased child autonomy) were associated with the use of CSE+F by 7 to 18 months of age.

Child screen exposure is common worldwide such that most children, regardless of parent, environment, or child characteristics, have screen exposure early in life as a potential cultural norm, even if current guidelines recommend against it.^29^ The high levels of CSE in the present study are mirrored by findings in several other studies that most children before age 2 years have considerable CSE, even increasing CSE over time.^16,42,43^ However, CSE+F in this age group appears to be less common.

Increased maternal distress and stress have been associated with increased CSE.^22,44^ We found that parenting stress as a particular type of distress was associated with increased CSE in this sample. Previous qualitative studies have identified individual stressors in parents’ motivations for CSE. In a study on parental screen use, some parents specifically cited CSE as a way to calm their children or prevent family conflicts.^28^ Another qualitative study on CSE identified parents’ view of CSE as a “safe and affordable distraction.”^4^^(p1303)^ Furthermore, parents indicated using CSE as a way to accomplish their own chores or tasks.^4^ For some families, CSE may act as a so-called digital playpen, particularly as children develop greater mobility and autonomy and cannot be constantly monitored while parents attend to other children, household responsibilities, or work obligations. Parents must often care for their children with little access to neighborhood resources, unsatisfactory partner engagement in childcare or household chores,^45^ or low extended family support, emphasizing parents’ need to keep a child entertained and contained with a “safe and affordable distraction.” As such, CSE may act as a peacekeeper not only among multiple children in the household but also among coparents, enabling them to avoid discord over inequitable sharing of childcare and household responsibilities. However, a 2014 study with children aged 2 to 5 years did not find parenting stress to be associated with CSE.^46^ Greater market penetration of smartphones, up from 56% in 2013 to 86% in 2018,^47,48^ may have made screen exposure easier for infants and toddlers compared with the environment in the 2014 study, particularly when combined with parenting stress. In short, parents may use CSE as a way to cope with individual stressors, such as family conflict, competing demands in the home, psychological distress, and parenting stress.

We found that factors in adverse child outcomes were also associated with CSE and CSE+F, indicating that the implications of CSE and CSE+F for child outcomes may be confounded by individual and contextual stressors. Previous research has demonstrated that television viewing in the context of lower available parental investment can have positive associations with adolescent test scores.^49^ Given the associations of individual and contextual stressors with adverse child outcomes,^50^ the associations of individual and contextual stressors with CSE and CSE+F, and the contingent associations between CSE and adverse child outcomes,^49^ we believe a contextualized approach is the most appropriate to understand family dynamics clinically and to develop productive research questions.

Although the studies on the associations between lower income and CSE have mixed results,^16,43^ the present study found that lower household income was associated with increased frequency of CSE, use of CSE+F, and increased frequency CSE+F. These associations may be owing to the unique pressures of having low income, such as neighborhood conditions and access to resources.^51^ As such, low income may limit actual or perceived options for CSE alternatives to occupy or entertain children.^4,28^ Parents may be using CSE and CSE+F to cope with contextual stressors, including lack of neighborhood resources or affordable and accessible alternatives to screen time, such as early childhood development centers, clean and age-appropriate playgrounds, or playgroups.^52^

### Limitations and Strengths

This study has several limitations. First, the sample consisted of relatively high-income, highly educated, mostly 2-parent families and may not be representative of all urban families, such as single parents managing multiple demands. Furthermore, most respondents were mothers, which does not capture how parenting stress may differentially affect fathers. Second, the self-reporting on variables may be biased by social desirability, such as parenting stress. However, we were still able to detect the associations of personal and contextual stressors with CSE and CSE+F in this sample with lower parenting stress and higher income.

Despite these limitations, this study also has several strengths. The study had an ethnically diverse and adequately powered sample, included detailed variables on CSE and CSE+F, considered several stressors, and used zero-inflated negative binomial analysis with parallel analyses of the occurrence of CSE or CSE+F and the degree of CSE or CSE+F. Although the variance explained by our model was small, the children were very young, and this analysis had a cross-sectional design. Systemic and cumulative effects with greater influence on later CSE, CSE+F, and associated outcomes may be present.^53^

## Conclusions

These results demonstrate the pervasiveness of CSE, with approximately three-quarters of children exposed to screens by 18 months of age and one-third exposed to screen time with food. Higher levels of parenting stress and lower household income were associated with the use of screens and a higher amount of daily screen time. Older child age and lower household income were associated with screen time while feeding, whereas lower household income was associated with more daily screen time combined with feeding. Given the neurodevelopmental health risks associated with early exposure to screen time and screen time combined with feeding, understanding why parents use screen exposure for young children is important to the development of successful interventions. Given that parenting practices develop early, interventions may include education about the risks of early screen time; alternative stress reduction and parenting strategies that do not involve screens; increased social support and resources within the home, such as coparenting; or more free community-based activities for parents and children.

## Notes

Supplement. **eTable 1.** Complete Descriptive Statistics on Missing and Complete Participants **eTable 2.** Correlation Matrix With Pairwise Deletion on Unimputed Data for All Participant Characteristics, Predictors, and Outcomes **eTable 3.** Number (%) of Participants Who Endorsed CSE by Modality and Year
