# Supplementary material for: Association of Parental and Contextual Stressors With Child Screen Exposure and Child Screen Exposure Combined With Feeding
Source: JAMA Netw Open. 2020 Feb 5;3(2):e1920557. doi: 10.1001/jamanetworkopen.2019.20557 (PMC12520712; doi:10.1001/jamanetworkopen.2019.20557)
Supplement: Supplement. — eTable 1. Complete Descriptive Statistics on Missing and Complete Participants eTable 2. Correlation Matrix With Pairwise Deletion on Unimputed Data for All Participant Characteristics, Predictors, and Outcomes eTable 3. Number (%) of Participants Who Endorsed CSE by Modality and Year [file jamanetwopen-e1920557-s001.pdf]

Supplementary Online Content

Tombeau Cost K, Korczak D, Charach A, et al. Association of parental and contextual stressors with child screen exposure and child screen exposure combined with feeding. *JAMA Netw Open.* 2020;3(2):e1920557. doi:10.1001/jamanetworkopen.2019.20557

eTable 1. Complete Descriptive Statistics on Missing and Complete Participants

eTable 2. Correlation Matrix With Pairwise Deletion on Unimputed Data for All Participant Characteristics, Predictors, and Outcomes

eTable 3. Number (%) of Participants Who Endorsed CSE by Modality and Year

This supplementary material has been provided by the authors to give readers additional information about their work.

eTable 1. Complete Descriptive Statistics on Missing and Complete Participants

Chi-square used for group (missing, complete) comparisons of binary variables. T-test was used for group (missing, complete) comparisons of continuous variables. * p < .05; ** p < .01

	**SCR**						**SCR+F**					
	**Missing (n = 362)**		**Complete (n = 723)**				**Missing (n = 275)**		**Complete (n = 810)**			
**variables**	**mean**	**%**	**mean**	**%**	**t or χ^2**	**p**	**mean**	**%**	**mean**	**%**	**t or χ^2**	**p**
Family living arrangements[1]	-	87.5	-	96.3	24.17	***< 0.01*** ***	-	85.5	-	96.0	30.18	***< 0.01*** ***
Relationship to child[2]	-	92.3	-	92.3	0.03	0.85	-	94.2	-	91.6	0.86	0.35
Mother Ethnicity[3]	-	43.6	-	58.2	18.49	***< 0.01*** ***	-	39.3	-	58.1	28.25	***< 0 .01*** ***
Mother age	34.15	-	34.52	-	-1.19	0.23	34.3	-	34.5	-	-0.52	0.61
Self-reported income[4]	-	66.6	-	89.1	14.75	***< 0.01*** ***	-	60.7	-	88.6	16.41	***< 0.01*** ***
Mother education level[5]	-	87.3	-	95.0	14.10	***<0.01*** ***	-	86.2	-	94.5	13.21	***< 0.01*** ***
Mother employment[6]	-	70.7	-	84.4	30.63	***< 0.01*** ***	-	74.4	-	84.0	33.09	***< 0.01*** ***
Other children in the home[7]	-	55.2	-	56.7	0.15	0.69	-	53.8	-	57.0	0.71	0.40
Child age[8]	-	68.5	-	72.9	2.06	0.15	-	68.0	-	72.6	1.90	0.17
Child biological sex[9]	-	52.7	-	52.3	0.03	0.86	-	53.1	-	52.2	0.08	0.77
IBQ Negative Affectivity	4.43	-	4.26	-	2.39	***0.02****	4.4	-	4.3	-	1.37	0.17
Total times child eats with screen in typical week[10]	5.89	47.51	3.86	64.9	3.75	***<0.01*** ***	5.9	47.6	4.6	64.4	2.90	***<0.01*** ***
Total screen time for the child in a typical week[11]	475.57	9.4	409.21	25.7	1.45	0.15	473.2	13.8	408.4	22.7	1.47	0.14
Parenting Stress Index total sum	62.71	-	61.53	-	0.92	0.35	61.9	-	61.8	-	0.09	0.93
1: % in 2-parent families; 2: % mother or step-mother; 3: % white or Caucasian; 4: % ≥ $100,000 per year, 5: % college-educated; 6: % employed; 7: % only child in home; 8: % 7-13 months of age; 9: % male; 10: % o screen food; 11: % 0 screen time												

©2020 Tombeau Cost K et al. *JAMA Network Open.*

eTable 2. Correlation Matrix With Pairwise Deletion on Unimputed Data for All Participant Characteristics, Predictors, and Outcomes

Generated in R with the *polycor* package to account for different data types (ordinal binary, continuous, categorical).
|0.10| > r < |0.30| is considered a small association; |0.30| > r < |0.50| is considered a medium association; r > |0.50| is considered a large association. Medium and large associations are identified with the correlation co-efficient in bold italics.

	**Characteristics**	**1**	**2**	**3**	**4**	**5**	**6**	**7**	**8**	**9**	**10**	**11**	**12**	**13**	**14**	**15**	**16**	**17**
**1**	Family living arrangements	1.00																
**2**	Relationship to child	-0.20	1.00															
**3**	Mother ethnicity	0.23	0.23	1.00														
**4**	Father ethnicity	***0.35***	-0.01	***0.78***	1.00													
**5**	Mother age	-0.13	-0.02	-0.13	-0.18	1.00												
**6**	Father age	-0.13	0.03	-0.03	-0.07	***0.62***	1.00											
**7**	Self-reported household income	***-0.52***	-0.10	***-0.46***	***-0.42***	0.28	0.20	1.00										
**8**	Mother education level	***-0.38***	-0.01	-0.18	-0.23	***0.33***	0.06	***0.56***	1.00									
**9**	Mother employed	0.28	0.24	***0.35***	0.29	-0.04	0.07	***-0.50***	***-0.42***	1.00								
**10**	Father employed	***0.32***	***0.32***	0.22	0.15	0.03	0.07	***-0.36***	0.07	0.20	1.00							
**11**	Other children in the home	-0.17	-0.13	-0.04	0.00	***0.30***	0.23	0.03	-0.08	0.23	-0.11	1.00						
**12**	Child age	-0.09	-0.15	-0.06	-0.05	-0.01	-0.05	0.23	0.10	-0.18	0.08	-0.09	1.00					
**13**	Child gender	0.00	0.07	-0.10	0.00	0.02	0.08	0.07	-0.04	0.00	0.04	0.00	-0.04	1.00				
**14**	IBQ Negative Affectivity	0.08	-0.02	0.19	0.18	0.00	0.03	-0.18	-0.03	0.10	-0.02	0.13	-0.13	-0.05	1.00			
**15**	Screen exposure combined with feeding	0.05	0.02	0.24	0.21	-0.09	-0.07	-0.23	-0.21	0.16	0.06	0.00	-0.15	-0.06	0.08	1.00		
**16**	Screen exposure	0.12	-0.16	0.08	0.13	-0.05	-0.01	-0.15	-0.11	-0.05	0.00	-0.07	-0.10	-0.05	0.04	***0.56***	1.00	
**17**	Parenting Stress Index total sum	0.19	0.03	0.08	0.11	0.00	0.03	-0.02	0.05	0.09	0.00	-0.05	-0.02	-0.03	0.22	0.00	0.07	1.00

©2020 Tombeau Cost K et al. *JAMA Network Open.*

eTable 3. Number (%) of Participants Who Endorsed CSE by Modality and Year

For each screen modality, percent calculations used the total number of participants who answered that specific question in that year as the denominator. For the total screen exposure across any types, percent calculations used the total number of participants who answered any question on screen exposure for that year as the denominator. Also, it is important to note that some participants endorsed multiple screen modalities for the same child.

Year	Awake in a room with television on?	Awake in a room with Videos or a DVD on?	Playing video game consoles?	Playing the computer?	Playing handheld devices?	Total across any screen types
	**n (%)**	**n (%)**	**n (%)**	**n (%)**	**n (%)**	**n (%)**
2011 (n = 6)	4 (66.67)	1 (16.67)	0 (0.00)	0 (0.00)	0 (0.00)	4 (66.67)
2012 (n = 70)	38 (55.88)	17 (24.64)	0 (0.00)	5 (7.25)	0 (0.00)	49 (70.00)
2013 (n = 54)	34 (69.38)	4 (8.00)	2 (4.00)	1 (2.00)	5 (10.00)	39 (78.00)
2014 (n = 130)	78 (66.67)	20 (18.18)	2 (1.85)	16 (14.16)	30 (26.79)	96 (73.85)
2015 (n = 297)	186 (68.13)	34 (13.99)	10 (4.13)	32 (13.17)	95 (38.00)	228 (76.77)
2016 (n = 401)	265 (69.55)	44 (12.98)	2 (0.59)	30 (8.88)	112 (32.18)	311 (77.56)
2017 (n = 114)	74 (68.52)	15 (14.42)	2 (2.00)	6 (5.94)	34 (33.33)	87 (76.32)
2018 (n = 13)	3 (25.00)	2 (18.18)	0 (0.00)	0 (0.00)	2 (18.18)	6 (46.15)
Total across all years	682 (67.26)	137 (14.72)	18 (1.96)	90 (9.68)	278 (29.36)	820 (75.58)[1]

1: includes participants who answered any question on any modality of screen exposure, which is why this n and percent are different than the percent with screen exposure in Table 1, which includes only participants who completed all questions on all modalities of screen exposure.

©2020 Tombeau Cost K et al. *JAMA Network Open.*
